# “Guidelines… yeah, they just haven’t felt relevant to me.” A qualitative exploration of chiropractors’ perspectives on physical activity promotion

**DOI:** 10.1177/10538127251350848

**Published:** 2025-06-19

**Authors:** Matthew Fernandez, Kathryn Di, Marina Pinheiro, Katie de Luca, Jeffrey Hebert, Peter Stilwell

**Affiliations:** 1 School of Health, Medical and Applied Sciences, CQUniversity, Brisbane, Australia; 2 Institute for Musculoskeletal Health, Sydney Local Health District, Sydney, Australia; 3 School of Public Health, Faculty of Medicine and Health, The University of Sydney, Sydney, Australia; 4 Faculty of Kinesiology, University of New Brunswick, Fredericton, Canada; 5 School of Allied Health, Murdoch University, Murdoch, Western Australia; 6 Movement, Culture and Society (MoCS), Department of Sports Science and Clinical Biomechanics, Faculty of Health Sciences, University of Southern Denmark, Odense, Denmark; 7 School of Physical and Occupational Therapy, Faculty of Medicine and Health Sciences, McGill University, Montreal, Canada

**Keywords:** physical activity, exercise, musculoskeletal, chiropractic, qualitative study, health promotion

## Abstract

**Background:**

Globally, almost one-third of adults are considered physically inactive. Chiropractors knownly promote physical activity (PA) within their musculoskeletal management plans, despite their limited PA and sedentary behavior (SB) guideline knowledge.

**Objective:**

To deepen our understanding of chiropractors’ perspectives, including factors that may influence PA promotion. Specifically our objectives are to (1) explore chiropractors’ knowledge of PA guidelines, (2) examine chiropractors’ practices in PA assessment and advice, and (3) identify barriers, enablers, and factors influencing PA promotion in chiropractic.

**Methods:**

Twenty registered Australian chiropractors were interviewed to understand their perspectives on promoting PA in practice. We used a qualitative descriptive approach with inductive content analysis to identify patterns and themes.

**Results:**

Four themes captured chiropractors’ perspectives regarding PA: (1) chiropractors striving to take a person-centered approach to PA promotion, (2) chiropractors had limited knowledge of the PA/SB guidelines, (3) chiropractors relied on their personal intuitions and experience to try and be PA role models for their patients, and (4) chiropractors identified important enablers including longer appointment time and patient motivation as well as barriers such as limited knowledge, skill and time. Chiropractors identified interest and motivation as patient barriers.

**Conclusion:**

Chiropractors have limited PA/SB guideline knowledge but nevertheless report being confident, safe and person-centered with respect to PA promotion, often relying on their own experiences to be PA role models for their patients. Supporting behavior change among chiropractors, while addressing time constraints and patient motivation are important considerations.

## Background

Described as any bodily movement produced by skeletal muscles requiring energy expenditure, physical activity (PA) has numerous health benefits, including the preservation of function and mobility, cardiorespiratory fitness, muscular strength, better mood, and cognitive function. In order to preserve and maintain these health benefits, the World Health Organisation (WHO) recommends 150 min of moderate or 75 min of vigorous activity per week, coupled with muscle strengthening.^1^ The WHO guidelines are inclusive of children over the age of five, older adults, pregnant and postpartum women, as well as individuals with chronic conditions or disabilities, and aim to reduce the burden of preventable conditions.^1^ Importantly, the WHO emphasizes that all PA, no matter the intensity or duration, provides health benefits.

However, despite these recommendations, almost a third of adults globally are insufficiently active,^2^ and physical inactivity is often described as a global pandemic.^3^ The individual and societal burden of physical inactivity has been extensively documented and includes substantial increases in the prevalence of non-communicable diseases,^1^ as well as direct health care costs estimated at more than $500 billion globally by the year 2030 if current trends continue.^4^ The WHO recognizes this societal challenge,^5^ and through its Global Action Plan on PA,^6^ endorsed a multi-level approach comprising active communities, environments, people, and systems^6^ to reduce non-communicable disease burden and improve population health.^2^ The Global Action Plan includes recommendations on active travel, urban design, and sport and recreation for all, with a call to action to drive PA into national and sub-national policies.^7^

Within the Global Action Plan, the healthcare sector is acknowledged as a key partner in promoting PA,^7^ where the healthcare sector includes allied health professionals like physiotherapists, podiatrists, exercise physiologists, and chiropractors – who consult with a large number of patients seeking health advice.^7^ Chiropractors are well placed to promote PA given their pattern of practice and focus on improving musculoskeletal health. Globally, a high percentage of chiropractors obtain information on PA behaviors from their patients and routinely counsel their patients with respect to PA.^8^ In Australia, 85% of chiropractors indicated they discuss PA promotion within their management plans,^9^ and despite their confidence in PA promotion, a recent national cross-sectional survey of chiropractors showed approximately 1 in 3 are ‘not at all familiar’ with PA and sedentary behavior (SB) guideline recommendations.^10^ Limited PA/SB guidelines knowledge is cited as a key barrier for healthcare professionals like physical therapists when implementing PA in practice, and they desired further training to promote PA within routine care.^11^ Chiropractors acknowledge the importance and assess or advise on PA, yet their knowledge of specific guidelines remains limited. This knowledge gap warrants further exploration by capturing practical insights into real-world challenges and understanding why such guidelines are overlooked and inform on further strategies or training for guideline implementation.

By expanding upon earlier research,^8–10^ the objective of this qualitative study was:

1. To explore chiropractors’ knowledge and understanding of PA guidelines;
2. To examine chiropractors’ practices in assessing and advising on PA; and
3. To identify barriers, enablers, and factors influencing PA promotion in chiropractic.

## Method

### Design

This qualitative descriptive study used semi-structured interviews to gain detailed insights into Australian chiropractors’ perspectives and practices regarding PA promotion. We used *qualitative description*^12,13^ as it is well suited for health research aiming to generate a straightforward summary of participants’ perspectives (i.e., knowledge, experiences, opinions, beliefs) and practices.^14–16^ This methodology categorizes participants’ self-reports and identifies common threads.^17^ Thus, qualitative description is appropriate for summarizing chiropractors’ perspectives and practices regarding PA promotion. Qualitative description is not highly interpretive and, therefore, does not require engagement with a specific conceptual or philosophical framework.^13^ This contrasts designs that use a particular theory (e.g., phenomenology) or aim to interpret data more abstractly to generate theory (e.g., grounded theory). Study reporting followed established qualitative research criteria - consolidated criteria for reporting qualitative research.^18^

### Ethics

Ethical approval for this study was obtained from the Human Research Ethics Committee at CQUniversity, Australia (reference number 0000024630).

### Setting and participants

We included registered Australian chiropractors in private practice. Chiropractors were recruited using purposeful sampling to obtain participants with diverse/varied backgrounds (e.g., variation in years of experience). Eligible chiropractors were recruited from February 20, 2024, for 3 months through advertisement using social media (Facebook) and professional associations (Australian Chiropractic Association, Chiropractic Australia). The advertisement briefly informed prospective chiropractors that the study would explore their perspectives on PA promotion to patients. Interested participants were emailed an invitation to participate and a consent form. Participating chiropractors received one $AU50 gift card. Recruitment of participants ended as soon as data saturation was reached, as outlined below.

### Sample size

We ceased recruitment and data collection, when saturation was considered reached and no new codes, themes, or meaningful variations were identified from successive interviews. The qualitative sample size of 20 was informed by prior methodological guidance,^16,19^ ensuring sufficient depth and breadth for meaningful analysis. Saturation was monitored through regular discussions among researchers (KD, MF, PS) and independent review of coded transcripts.

### Data collection

Participants were initially asked to provide demographic data (gender, years of practice experience, type of practice setting, Likert scale for PA promotion status (‘never’, ‘rarely’, ‘sometimes’, ‘often’, and ‘all the time’), State/Territory, and exercise science or health promotion education (workshop, diploma, degree).

An interview guide was designed based on the results of a recent systematic review^8^ and previous quantitative studies related to PA promotion in chiropractic.^9,10^ All co-authors reviewed the guide, which was pretested by team members (KD, MF) through a mock interview and debriefing with an experienced qualitative researcher (PS). As a result, the interview guide was slightly adapted. All but one interview was led by one co-author (KD). The lead author (MF) and KD conducted the first interview together. Both researchers (KD, MF) had relevant interview experience. Interviews took place on an online platform (Zoom) and were approximately 15–25 min in duration, audiotaped and professionally transcribed (https://waywithwords.net/). The guide included nine interview questions (Appendix), targeting (1) PA definitions, (2) advising or discussing PA in practice, (3) knowledge of the PA/SB guidelines, (4) PA information routinely gathered from patients, (5) perceived barriers and enablers to PA promotion, (6) skills and training needed to promote PA and (7) chiropractors own PA influencing practice and whether they meet the PA/SB guidelines. Participants were given the opportunity to review their transcripts for accuracy and make any necessary edits to ensure the text reflected their intended responses.

### Data analysis

We conducted an inductive (conventional) qualitative content analysis^20,21^ of the transcripts, during the ongoing data collection and analysis, to identify patterns and generate themes (i.e., categories summarizing participants’ responses). Content analysis is recommended for qualitative descriptive studies.^13,19^ To manage and code the interview data, we used Microsoft Excel. Initially, three researchers (PS, KD and MF) reviewed and coded data from one transcript to establish a consistent approach to coding. The remainder of the coding was completed by two researchers (KD and MF), who each reviewed a subset of transcripts independently. Through regular meetings, KD and MF ensured coding consistency and generated preliminary themes that were further refined based on input from PS. To enhance rigor, PS audited three transcripts to ensure the themes accurately reflected the data. Subsequently, the full research team provided feedback to generate the final themes.

## Results

We interviewed 20 chiropractors, and their demographic characteristics are presented in Table 1. Briefly, 65% of the sample were men and 85% worked in private practice. Forty-five percent had less than 5 years of clinical experience, while 45% had more than 20 years of practice. Three-quarters (75%) practiced in Queensland and New South Wales, and 95% of chiropractors often-to-always promoted PA in their clinical setting.

**Table 1.** Physical activity – demographics of participating Australian chiropractors.

		N	%
Total		20	
Gender	Male	13	65
	Female	7	35
Years practicing chiropractic	0–5	9	45
	6–10	0	0
	11–15	1	5
	16–20	1	5
	20+	9	45
Highest level of education	Diploma	1	5
	Bachelor's degree	4	20
	Masters	12	60
	Other (Doctor of Chiropractic)	1	5
	Doctorate (PhD)	2	10
Work setting	Private Clinic	17	85
	Academic (e.g., teaching/supervision)	1	5
	Retired	0	0
	Private practice and academic	2	10
Location	QLD	9	45
	NSW	6	30
	VIC	4	20
	WA	1	5
	SA	0	0
	TAS	0	0
	NT	0	0
	ACT	0	0
Promotion of physical activity in a clinical setting	Never	0	0
	Rarely	0	0
	Sometimes	1	5
	Often	9	45
	All the time	10	50

N: number; %: percentage; QLD: Queensland; NSW: New South Wales; VIC: Victoria; SA: South Australia; TAS: Tasmania; NT: Northern Territory; ACT: Australian Capital Territory.

### Themes

**Figure 1.**
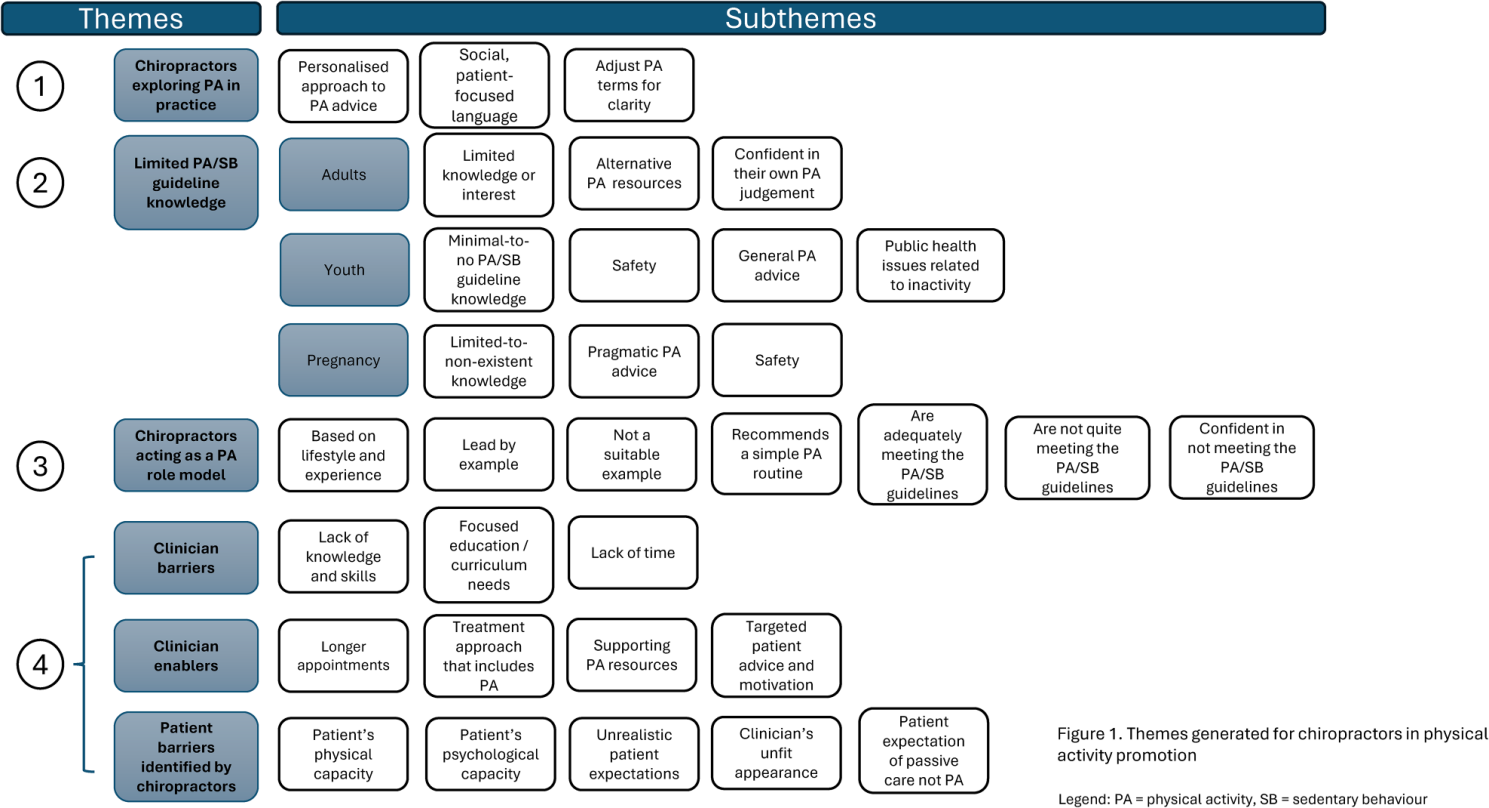
Themes and subthemes.

We generated four themes from the data: (1) Chiropractors report person-centered PA promotion, (2) chiropractors have limited PA/SB guideline knowledge, (3) chiropractors want to be PA role models and (4), barriers and enablers to PA promotion. Figure 1 displays the four themes identified in the study, along with the corresponding sub-themes discussed within each.

#### Theme 1: chiropractors report person-centered PA promotion

Chiropractors report that they take a personalized approach when assessing their patients physical capacities and when advising and/or discussing PA. This included evaluating their patients’ injuries, ability to move, and other health-related issues that may impact PA, ensuring that any PA recommendations are not only physically appropriate but also contextually meaningful. Chiropractors also reported considering their patients preferences, hobbies, and how they build upon and complement what their patients are already doing, reflecting a philosophy that values the holistic well-being and patient's autonomy.

“*(I assess) current limitations … in terms of their ability to move … any injuries they may have, that sort of stuff. What they’re telling me … is most important.” (Chiro 4)*

“*I want to know what they generally do, what they’ve been doing, and what they enjoy doing … it has to be doable for them” (Chiro 15)*

Chiropractors also reported that they try to use person-centered language when discussing PA, emphasizing individuality, using encouraging and relatable terms such as “fun” and “social”. This highlights that chiropractors frame PA to resonate with patients’ values. Additionally, chiropractors leaned towards the term “movement” suggesting a more functional orientation and how it can be enjoyable to increase daily activity capacity and to produce “feel-good” hormones:

“*Movement that is enjoyable for them and produces serotonin and good feelings.” (Chiro 10)*

Chiropractors were conscious of the terms “physical activity” and “exercise”, and appeared to customize their language to their patients to enhance their understanding:

“*I don’t know what a patient would understand when I say, I need you to do more physical activity… But if I said, I want you to do some exercise … I feel like they would intuitively understand what that means.” (Chiro 2)*

#### Theme 2: chiropractors have limited PA/SB guideline knowledge

Most participants reported limited knowledge or interest in PA/SB guidelines and relied on alternative rationales or sources, often deferring to their own experience or professional colleagues. Nevertheless, the lack of guideline knowledge did not dampen chiropractors’ confidence in prescribing PA, relying on their own judgement, i.e., any movement is better than nothing, to inform patients. Chiropractors confidence appears to stem from practice-based reasoning rather than evidence-based guidelines.

“*I don’t know what they are exactly* *…* *I haven’t ever physically sought them out* *…* *I'll jump on to Google if I was curious about them exactly.” (Chiro 12)*

“*30 min of moderate to vigorous activity a day. I couldn’t tell you where it’d came from. Um, yeah, I think any form of movement is better than none. So, if you can get at least a 20-min walk or some type of physical activity in, it’d be more beneficial than none. But I couldn’t tell you which guideline that comes from.” (Chiro 3)*

Some participants were able to name specific guidelines or touch on recommendations for adults, but less so for youth, or pregnant women. Regarding Australian PA/SB guideline recommendations for adults, some chiropractors acknowledged other recommendations that contribute to PA levels, like completing 10,000 steps per day, or activities that make their patient ‘huff’ and ‘puff’ a few times per week (and gradually progress it). For youth populations (children and adolescents), chiropractors overall demonstrated minimal-to-no PA/SB guideline knowledge:

“*I’ll be honest with you, possibly (I’ve) looked at it (guidelines), and then moved on, so not much.” (Chiro 8)*

However, chiropractors regarded safety as an important feature of youth PA participation. This included caution with over activity; children and adolescents having symptoms and having to hold them back from PA. This underscores a protective, risk-aware stance, relfecting a cautious approach:

“*Kids … are ridiculously active, managing (their) activity levels when they irritate things.” (Chiro 13)*

Further, despite the lack of knowledge, chiropractors did make non-specific (or general) PA recommendations for youth populations, like getting outside, moving and playing due to public health issues like increasing screen time and obesity:

“*I haven’t looked up kids* *…* *(but) we have a growing problem of obesity* *…* *I teach people about the need for, at every age level, to be moving.” (Chiro 18)*

For pregnant women, PA/SB guidelines knowledge was evident in some chiropractors who acknowledged it was the same as the adult population. However, for others, knowledge was very limited-to-non-existent:

“*Nothing at all. I mean, I probably have read about them. I’ve probably heard about them. And again, I haven’t stored it in my brain. I don’t have it stuck on my wall in my room.” (Chiro 2)*

Nevertheless, there was evidence of providing general advice for PA regardless of their guideline knowledge, and that chiropractors were advocates for a safety-first approach, ensuring modifications so PA for pregnancy is of lower intensity, preventing overheating and pelvic instability. This emphasizes adaptation and risk minimization over the general promotion of PA.

“*I encourage them (pregnant women) to stay fit and active and all of that. But I can’t say I’ve got guidelines.” (Chiro 20)*

“*For example, no handstands, no box jumps, because they start becoming a matter of safety, obviously.” (Chiro 4)*

#### Theme 3: chiropractors want to be PA role models

Chiropractors’ own PA journey and experiences appear to significantly shape their PA promotion in their clinics and their desire to be role models to their patients. They report to ‘lead-by-example’ or ‘walk the talk’ and that their PA promotion is based on their own active lifestyles, personal experiences, and their understanding of health:

“*The more active and the more benefits you can see yourself, then the more likely you are to recommend it, things like that. But it's also a bit of a lead-by-example.” (Chiro 7)*

Specifically, through their own explorations or new (physical) activities, chiropractors bring examples into their conversations with patients:

“*Actually, I’m really sore, because I just ramped up some resistance work* *…* *affects me in practice, like, ooh, well I’m a little sore* *…* *I pushed the chest stuff the other day.” (Chiro 13)*

Conversely, some chiropractors felt their PA experiences were not suitable for their patients, often referring to their own physical condition:

“*If I was in a better fitness state myself; I would probably hold my patients to a better standard maybe.” (Chiro 20)*

In terms of personal PA participation by chiropractors, they reported keeping it simple. Walking was the most cited activity, followed by gym-based activities:

“*I love a walk, daily occurrence* *…* *I’ve got weights, I do a bit at home.” (Chiro 18)*

For others, exercise dose was important, as was their motivation that triggered high amounts of PA participation. Along these lines, chiropractors (overall) felt that they were meeting PA/SB guidelines. They appeared confident in this respect, particularly when they were knowledgeable about the guidelines. This appears to reinforce their credibility and alignment with the advice they offer patients:

“*Yes, above and beyond (guideline recommendations), probably.” (Chiro 17)*

However, there was a hint of inconsistency, with chiropractors either barely or not quite reaching the desired PA/SB level recommended, based on their limited PA/SB guideline knowledge:

“*I could do better myself, absolutely. I would come, kind of, close, but no, not every week.” (Chiro 11)*

For other chiropractors, the uncertainty was clear, on the back of being unsure or not at all familiar with the guidelines. This points to a potential gap between professional advice and personal practice:

“*I probably am not meeting, you know, the moderate intensity (guidelines) and all that sort of stuff.” (Chiro 20)*

#### Theme 4: barriers and enablers to PA promotion

This section addresses clinician barriers and enablers and concludes with patient barriers identified by the chiropractors.

##### Clinician barriers

For participating chiropractors, the perceived lack of knowledge and skills to implement PA was evident. For instance, fear of injuring their patients by providing advice they were not sure of, thus putting the patient at risk and re-aggravating them. Level of knowledge and skills were related to education and culture. For instance, being a recent graduate (chiropractor) was considered advantageous to an ‘old-school’ or ‘traditional’ clinician, possibly due to PA advancements included within contemporary curriculums, like rehabilitation and behavior change techniques. This suggests recent gradulates may enjoy an advantage in understanding and applying current PA practices:

“*There was a pretty big component about rehabilitation in particular* *…* *I reckon we’re fairly well educated already to be able to support patients with our knowledge base that we’ve got already.” (Chiro 19)*

For other chiropractors, there was recognition of gaps in knowledge and a desire for professional growth, through a perceived need to upgrade their qualifications and competencies. For instance, from a diagnostic orthopedic assessment to a more ‘real-world’ functional assessment, or further personal training studies to gain more exercise prescription skills. Other chiropractors targeted education, specifically Accreditation Bodies and Universities to focus on new PA knowledge:

“*The problem I see, is that, that our accreditation bodies are based on a lot of knowledge that was, was amassed quite some time ago…” (Chiro 9)*

Practical constraints like time were also considered a barrier. Participants often commented on the practices of other chiropractors to emphasize how time constraints are shaped by the clinic or the chiropractor's philosophy or approach to care, suggesting organizational and personal approaches to practice impact time management:

“*If they’re seeing people in, you know, two seconds, just ripping through these clients and not having a conversation with them. Maybe a time thing.” (Chiro 8)*

“*(The) approach that a clinician will do is very passive, so it actually just discourages the person to do anything because … They’ve been told, hey, I’ll do it for you.” (Chiro 6)*

##### Clinician enablers

For enablers, longer appointment time was an important factor. Clinicians who have longer appointments reported that they can include PA promotion:

“*I’ve always carved out a little bit of time for exercise prescription* *…* *those who have shorter appointment windows would potentially struggle, I guess.” (Chiro 12)*

Enablers also included chiropractor's overall treatment approach, including their use of resources that they could direct their patients to (e.g., printed materials, Apps, website links) and their ability to plan and provide targeted advice and motivational support. This reflects an adaptive approach to support and enhance patient engagement in health management:

“*Get them to walk for five minutes or get them to walk just before that point where they're fatigued, and slowly build up their tolerance.” (Chiro 14)*

“*(It's) all about having a plan and putting that plan together.” (Chiro 6)*

##### Patient barriers

Participating chiropractors commented on physical and psychological patient factors that they believe are important barriers to PA promotion. For instance, participants commented on their patients’ weight, health conditions, age, and lack of interest in PA – making it hard to promote PA. Overall, these highlight the multifaceted challenges in encouraging patient participation in PA:

“*It's just people with barriers in their brain. I don’t like exercising. I don’t like to sweat. Or whatever. Or little things like people don’t want to go to a gym because they don’t want to be seen by other people.” (Chiro 2)*

“*Like physical ailments and ageing bodies and arthritis and heart disease and, yeah, all of those kinds of things (are barriers).” (Chiro 2)*

Chiropractors specifically emphasized patients’ perceptions and expectations as barriers. For instance, they felt that if patients don’t see any benefit or results, or if they don’t have goals, they aren’t going to engage in PA. Furthermore, participating chiropractors believed that patients judge clinicians and question the credibility of their advice:

“*As horrible as it is, like, people look at clinicians and physios and, you know, doctors or PTs of certain weight and if they’re promoting physical activity, they tend to sort of skew towards if they look like they actually exercise.” (Chiro 4)*

“*Patients will sometimes use a practitioner's lack of visible fitness as an excuse, saying “If you can't do it, why should I?” (Chiro 9)*

Participating chiropractors also felt that important barriers included patients’ expectation of passive care and PA not being a priority nor what they want. Chiropractors consider their patients to be less interested in PA when they come to the clinic. Patients either perceive a personal trainer or a physiotherapist for exercise and they only seek passive care from chiropractors. This suggests a disconnect between patient preferences and active health promotion:

“*When I give, like, physical activity, they’re like, oh, I’ve never had that at a chiropractic appointment before, usually that's, like, a physio thing.” (Chiro 5)*

Equally, there is a perception by the patient that chiropractic adjustments promote overall wellness and address a wide range of issues**:**

“*The adjustment will be the thing that magically fixes everything, then that's a problem.” (Chiro 18)*

## Discussion

To our knowledge this is the first qualitative exploration of Australian chiropractors’ perspectives in the PA promotion space. Chiropractors appear to take a personalized approach to PA promotion, despite having limited knowledge of PA/SB guideline recommendations. Further, chiropractors confidently relied on their own experiences and alternative resources to promote PA. Noted barriers to PA promotion included time limitations, minimal PA knowledge and skill, while enablers consisted of having adequate time during appointments, and their ability to provide targeted advice and motivation support. Patient barriers related to their PA interest and motivation.

### Guideline knowledge

Knowledge and awareness of the PA/SB guidelines is arguably important to effectively recognize and promote PA. The limited guideline knowledge observed within our 20 participants fits closely with our previous findings, where approximately 40% of Australian chiropractors were ‘not at all familiar’ with national PA/SB guidelines.^10^ Notwithstanding, these findings are similar to other health care professionals (HCPs).^22,23^ For instance, PA promotion uncertainty among Australian podiatrists was attributed to insufficient knowledge of current guidelines.^23^ However, the lack of guideline knowledge did not appear to impact chiropractors’ confidence in discussing PA, particularly with the adult population. This is not only consistent with the WHO slogan: ‘Every Move Counts’,^1^ but aligns with the literature, particularly among physicians and psychologists, who were confident in their ability to promote PA.^24^ Further, Australian physiotherapists consider themselves well-equipped to promote non-treatment PA despite limited guideline knowledge.^22^ Collectively, findings suggest that guidelines are not widely used to inform clinical practice, potentially due to a lack of perceived relevance.^25^ This topic should be further explored, paving the way for deeper insights. For instance, it is presently unclear whether a lack of explicit guideline knowledge affects the application, safety or health outcomes associated with PA promotion.

Consistent with prior research that showed chiropractors were less likely to promote PA to youth and pregnant populations,^10^ chiropractors in our study demonstrated more limited PA/SB guideline knowledge for these populations (compared to adults). While chiropractors were aware of the PA benefits for youth and pregnant women, knowledge of the PA/SB guidelines remains limited. Similar findings have been noted with other HCPs. For example, a survey of midwives showed they were not confident guiding pregnant women on how to exercise, with more training preferred to effectively guide PA.^26^ Knowledge gaps have been found with other HCPs managing pediatric diabetes; with one in five senior doctors and specialist nurses citing insufficient knowledge to provide PA support to children and adolescents.^27^ Even if chiropractors are familiar with the PA/SB guidelines, without knowing how to implement them, progress may be limited. Further investigation should consider the implementation of educational resources that empower clinicians to apply the PA/SB guidelines.

### Inspiring PA as role models

Consistent with other HCPs,^22^ chiropractors in our study reported that their own personal life experiences shaped how they promoted PA. Strong (personal) motivation appeared to dictate their PA participation – from their active lifestyle, experience and/or individual goals. Taking a personalized approach to PA is a key strength supported by a recent review showing that HCPs were motivated to maintain their own health and prevent disease as a professional obligation (i.e., to lead by example). This, in turn, transfers PA skills and knowledge from their own lived experiences,^28^ such as engaging in a particular sport, to their interactions with patients.^29^ This raises the question about the potential need for future training and support within the clinical or exercise-promoting environment, such as real-time patient interactions or hands-on workshops, to better frame patient expectations, rather than in a formal learning or classroom setting.^22^ Chiropractors in our study showed variety in their own PA participation, from walking to significant levels of PA engagement. This is largely consistent with literature demonstrating that HCPs who engage in moderate-to-high PA levels, become influential advocates,^30^ enabling them to connect with patients when promoting PA.^28^

Equally, while chiropractors’ PA knowledge gained through their personal experiences can shape their role as models of healthy behavior, they need to be conscious that not all patients may necessarily be influenced by their personal standards. Patients may not be ready (i.e., stages of behavioral change) or even want to meet suggested standards. This can misalign with patient-centered care or advice reported by chiropractors in our study, unintentionally imposing their own exercise routines or beliefs. Chiropractors may therefore benefit from additional training in multiple behavior change strategies^11^ to better support patients in a way that aligns with their individual readiness, needs, and context.^31^ This further includes demonstrating empathy, offering unconditional positive regard, and maintaining a genuine commitment to addressing the patient's healthcare needs.^32^

### Enablers for chiropractors

Having sufficient time through extended appointments to develop rapport with patients during the clinical encounter was a noted enabler of PA promotion. Like other HCPs, chiropractic care lends itself to repeat opportunities for ongoing PA conversations,^33^ and therefore, chiropractors see PA promotion aligned with their clinical role in supporting overall health beyond addressing specific injuries or conditions.^22,33,34^ Further, supporting resources that are easily accessible in a patient-friendly (informational) format help simplify the process of introducing and communicating the PA message.^24,35^ While written materials were commonly adopted,^24^ the visual appeal of infographics can be easily shared across various digital/online platforms, making them more accessible and engaging for patients.^36^

Patient motivation and acceptance of PA were also an important enabler. This centered around chiropractors focusing on patient activities already underway, then building a plan around that activity, and having someone (i.e., the chiropractor) serve as an accountability partner. Chiropractors essentially adopted elements in line with that of a coaching role.^37^ Traditionally, enablers of exercise or PA promotion by HCPs have received limited attention in the literature.^38^ However, in response to patient's interest in exercise and motivation to change their behavior, HCPs like physiotherapists knowingly tailor their approaches to foster ongoing patient engagement in PA.^37^ Given that patients value the advice of HCPs,^24^ a person-centered approach can tie effective behavior change techniques, such as goal setting and self-monitoring, to achieve longer-term increases in PA.^39^ Therefore, behavior change techniques are an important knowledge target and should be considered for practical implementation when promoting PA.^40^

### Barriers for chiropractors

Chiropractors in our study were of the view that the recent graduates are ‘more up to date’ with greater knowledge and skill in PA promotion than their older or more experienced colleagues. While this may be attributed to exercise prescription (e.g., specifically designed exercise plans) being commonly recommended for patients within current chiropractic curriculums,^41^ this rationale also aligns with a recent review citing clinicians’ poor knowledge and confidence promoting PA as well as current practice workloads as key barriers to PA promotion.^24^ For these reasons, it is plausible that chiropractors would not prioritize initiating PA-related conversations.^31^

Increased knowledge and skills could facilitate greater confidence and support PA counseling.^11^ However, an underlying challenge may be chiropractors’ apparent lack of understanding or acknowledgement regarding the relevance of PA/SG guideline recommendations. As such, action is needed both at the university level and through external providers to increase awareness of the PA guidelines.^34,35,42^ At the very least, a concerted effort to consolidate PA information (i.e., core principles) through specific social media links, professional body magazines, websites, and accompanying material like visual infographics and leaflets in one place may increase awareness and implementation of the PA guidelines for both patients and chiropractors.^34,43,44^ Importantly, there is a need to better integrate the PA message into clinical practice in ways that are suitable to people living with clinical conditions.^45^ These actions could facilitate the personalized approach identified by chiropractors in this study.

A lack of time for PA promotion was a common barrier. This is in line with a recent review that acknowledges time as clinicians’ greatest limitation.^11^ Given the multiple tasks and priorities involved with managing health conditions, providing meaningful PA advice in a short time frame is challenging.^46^ However, qualitative data from U.S. physical therapists noted that they often find opportunities to encourage PA when providing manual therapy or exercise interventions, thus including brief interventions into practice without requiring additional time.^31^ Hence, there is potential to train and address skills that allow brief PA advice to be integrated into practice despite time constraints.^47^

### Patient barriers identified by chiropractors

The absence of patient interest and motivation was a common barrier to PA promotion reported by chiropractors, and consistent with literature suggesting German physiotherapists may quit PA promotion when faced with resistance or lack of patient engagement.^38^ This finding is also noted with Australian physiotherapists and podiatrists, who encountered difficulties motivating their patients for PA^22,23^ and is likely to reduce HCP confidence in promoting PA with their patients.^48^ Targeted training in motivational interviewing, behavior change strategies, and confidence-building techniques are needed to help clinicians better collaborate with patients to promote PA effectively.

### Strengths and limitations

Strengths of our study included the adoption of a purposive sample of chiropractors with different levels of professional experience. Thus, our interviews provided diverse information regarding clinicians’ opinions and approaches. Our data analysis involved multiple steps to increase rigor, including independent coding and auditing. Our virtual interviews enabled the recruitment of chiropractors nationwide, and by providing detailed context, location, and an in-depth description of the studied population, readers can assess how transferable the findings are to a similar setting. It is important to note, however, that in our study chiropractors were clustered in specific regions (primarily the east coast of Australia) and we did not aim to generalize our findings to all chiropractors across all of Australia. We also acknowledge other limitations in our study, that include elements of selection bias are associated with chiropractors possibly altering their responses to align with perceived social norms or expectations, or chiropractors who may have been more enthusiastic about the PA topic. This may potentially overstate the general role of PA in chiropractic practice. We did not stratify by variables such as gender, years of practice, or region, as our goal was to provide a broad descriptive account of participants’ perspectives. This could be explored in more targeted research. Lastly, we did not capture how PA promotion is perceived by those who are directly impacted - the patients. The literature indicates that Australians physiotherapists are expected, by their patients, to offer guidance on PA and general health behaviors,^49^ hence it should also be explored as to whether chiropractors should also anticipate that patients expect such advice.

### Clinical and research implications

Despite demonstrating PA awareness and confidence, efforts are required at both the university level and through targeted continuing professional development, for sustainable knowledge enhancement and implementation of PA/SB guidelines recommendations.^24^ One targeted, strategy to address the knowledge gap is to incorporate an actionable plan into existing clinical workflows that make PA a routine part of the treatment for musculoskeletal pain.^50^ For instance, a visual roadmap that encourages achievable light-intensity PA - such as taking brief walks - while promoting the immediate benefits of PA, like the possibility of temporary pain relief, improved mood, stress reduction, and a boost in self-confidence.^50^ Here, chiropractors can provide sufficient information and support while actively working to overcome obstacles to recovery, e.g., patient apprehension about PA due to potential reinjury.^51^ These efforts should consider the role of programs that build confidence in the chiropractor's ability to deliver basic and effective behavioral change techniques skillfully.^52^ Other targets for skill development include patient-centred motivational interviewing or brief counseling to overcome the lack of time as a barrier. Further, identifying stages of readiness for change along with goal setting and social support, using respectful, non-judgmental language to help guide the development of interventions to improve and sustain PA engagement. Additionally, the importance of patient-centered communication arises as chiropractors often rely on their personal intuitions and strive to be role models for PA to their patients. Finally, given chiropractors’ expressed willingness to promote PA despite knowledge-based barriers, their engagement with national professional associations may serve as a medium for influencing policy and expanding chiropractors’ roles within PA promotion frameworks. Integrating chiropractors more formally into public health initiatives - through clearer inclusion in interdisciplinary care models and expanding healthcare coverage - could help operationalize their potential contribution in both private and public settings. These multifaceted efforts could provide the necessary support to bridge the gap between knowledge and practice.

Chiropractors should recognize and address their biases to mitigate the challenge of imposing their personal perspectives on their patients, who may have different goals, values, and circumstances.

## Conclusion

Chiropractors have limited PA/SB guideline knowledge but nevertheless report being confident, safe and person-centered with respect to PA promotion, often relying on their own experiences to be PA role models for their patients. In light of the noted barriers and enablers to PA promotion, there is scope for improvement within the profession through university and post-graduate continuing education programs that train chiropractors, particularly to facilitate the use of evidence-based behavior change techniques. Additionally, strategies that address challenges such as time constraints and patient motivation are equally important.

## Supplemental Material

sj-docx-1-bmr-10.1177_10538127251350848 - Supplemental material for “Guidelines… yeah, they just haven’t felt relevant to me.” A qualitative exploration of chiropractors’ perspectives on physical activity promotion Supplemental material, sj-docx-1-bmr-10.1177_10538127251350848 for “Guidelines… yeah, they just haven’t felt relevant to me.” A qualitative exploration of chiropractors’ perspectives on physical activity promotion by Matthew Fernandez, Kathryn Di, Marina Pinheiro, Katie de Luca, Jeffrey Hebert and Peter Stilwell in Journal of Back and Musculoskeletal Rehabilitation

sj-docx-2-bmr-10.1177_10538127251350848 - Supplemental material for “Guidelines… yeah, they just haven’t felt relevant to me.” A qualitative exploration of chiropractors’ perspectives on physical activity promotion Supplemental material, sj-docx-2-bmr-10.1177_10538127251350848 for “Guidelines… yeah, they just haven’t felt relevant to me.” A qualitative exploration of chiropractors’ perspectives on physical activity promotion by Matthew Fernandez, Kathryn Di, Marina Pinheiro, Katie de Luca, Jeffrey Hebert and Peter Stilwell in Journal of Back and Musculoskeletal Rehabilitation
