# Supplementary material for: “Guidelines… yeah, they just haven’t felt relevant to me.” A qualitative exploration of chiropractors’ perspectives on physical activity promotion
Source: J Back Musculoskelet Rehabil. 2025 Jun 19;39(1):142–53. doi: 10.1177/10538127251350848 (PMC12783367; doi:10.1177/10538127251350848)
Supplement: sj-docx-1-bmr-10.1177_10538127251350848 - Supplemental material for “Guidelines… yeah, they just haven’t felt relevant to me.” A qualitative exploration of chiropractors’ perspectives on physical activity promotion [file sj-docx-1-bmr-10.1177_10538127251350848.docx]

**APPENDIX – interview guide / questions**


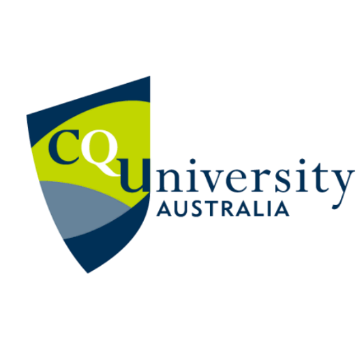


All interviews will be carried out by the primary researcher / research assistant who will have relevant interview skills. The interview (guide) will be structured as follows:

Hello, welcome to our qualitative study: making sense of physical activity promotion in chiropractic. Thank you for joining me. I’d like to commence with key definitions in relation to physical activity and exercise.

- Physical activity is any bodily movement.
- Exercise is a plan of activities.
- Exercise therapy is a plan of activities designed for rehabilitation.

1. Based on the definition above, in practice would you use the term exercise or physical activity (or other), and what is the rationale or reason for doing so?
   - Rationale – not all chiropractors are familiar with PA/SB guidelines, thus the term exercise may come more naturally and possibly from a rehabilitative perspective (i.e., exercise therapy), as opposed to general day-to-day movement (physical activity).
2. How would you discuss physical activity in routine practice? Follow up: How often do you discuss physical activity?
   - Rationale – 90% are prepared and routine discuss / advise / counsel on PA based on our review.
3. Can you tell me what you know about the physical activity and sedentary behaviour guidelines for adults – from any specific resource or guideline? Alternatively, what do you know about the WHO or the Australia physical activity and sedentary behaviour guidelines for adults?
   - Rationale - 1 in 3 chiropractors are not at all familiar with the guidelines.
4. Can you tell me what you know about the physical activity and sedentary behaviour guidelines for children and adolescents – from any specific resource or guideline? Could you tell me a bit more about whether you advise or discuss physical activity recommendations for children and adolescents?
   - Rationale - chiropractors (in Australia) who frequently promote physical activity as less likely to do so to children and adolescent populations.
5. Can you tell me what you know about the physical activity and sedentary behaviour guidelines for pregnant women – from any specific resource or guideline? Could you tell me a bit more about whether you make physical activity recommendations for pregnant women?
   - Rationale - chiropractors (in Australia) who frequently promote physical activity as less likely to do so to with pregnant women.
6. What information do you consider pertinent (relevant) that patients provide you in order to promote physical activity?
   - Rationale – a high percentage of chiropractors ask and gather physical activity participation information from their patients.
7. This next Q looks at what helps/hinders PA.

What do you feel are the perceived barriers and enablers for chiropractors to promote physical activity in practice? Follow up: What are your personal barriers and enablers, in promoting physical activity in practice?

- - Rationale – most cite (1) time as a barrier followed by (2) a lack of reimbursement and (3) a belief that behaviour change for physical activity will not occur.

1. What do you feel are the skills and training needed to help or more easily promote physical activity in chiropractic practice – can you tell me about what types of support tools do you feel are required?
   - Rationale – to inform a future educational tool kit and/or interventional study. Considering the reported limited PA knowledge/skills among chiropractors e.g., further training/education in this field has been identified.
2. What does your own physical activity participation consist of? Do you feel you (as the chiropractor) are meeting the physical activity and sedentary behaviour guidelines? Also, tell me about whether your own activity participation influences how you promote physical activity in practice?
   - Rationale - >75% of chiropractors report actively meeting the PA/SB guidelines (aerobic and resistance training).

Given the semi-structured nature of the interview guide, interviewers have flexibility to probe participants to elaborate on specifics provided in their responses. Questions will aim to explore the content of physical activity delivered/advised etc. during the consultation, or any concerns that arose during the consultations.

**Probing questions**

- Could you describe that in more detail?
- What did you think then?
- How did you feel then?
- What do you think it was due to?
