# Supplementary material for: “Guidelines… yeah, they just haven’t felt relevant to me.” A qualitative exploration of chiropractors’ perspectives on physical activity promotion
Source: J Back Musculoskelet Rehabil. 2025 Jun 19;39(1):142–53. doi: 10.1177/10538127251350848 (PMC12783367; doi:10.1177/10538127251350848)
Supplement: sj-docx-2-bmr-10.1177_10538127251350848 - Supplemental material for “Guidelines… yeah, they just haven’t felt relevant to me.” A qualitative exploration of chiropractors’ perspectives on physical activity promotion [file sj-docx-2-bmr-10.1177_10538127251350848.docx]

COREQ (COnsolidated criteria for REporting Qualitative research) Checklist PA

A checklist of items that should be included in reports of qualitative research. You must report the page number in your manuscript where you consider each of the items listed in this checklist. If you have not included this information, either revise your manuscript accordingly before submitting or note N/A.

| **Topic** | **Item No.** | **Guide Questions/Description** | **Reported on**  **Page No.** |
| --- | --- | --- | --- |
| **Domain 1: Research team**  **and reﬂexivity** | | | |
| *Personal characteristics* | | | |
| Interviewer/facilitator | 1 | Which author/s conducted the interview or focus group? | KD |
| Credentials | 2 | What were the researcher’s credentials? E.g. PhD, MD | Master’s and PhD |
| Occupation | 3 | What was their occupation at the time of the study? | Research assistant (CQU), post doctorates (McGill, USYD), senior lecturer (CQU), associate professor (CQU) and professor (New Brunswick) |
| Gender | 4 | Was the researcher male or female? | 3 males and 3 females |
| Experience and training | 5 | What experience or training did the researcher have? | All researchers have professional degrees in chiropractic, exercise science and physiotherapy. Five of the six have PhDs with extensive research experience. |
| *Relationship with*  *participants* | | | |
| Relationship established | 6 | Was a relationship established prior to study commencement? | No relationship was established with the participants prior to the study commencing. |
| Participant knowledge of  the interviewer | 7 | What did the participants know about the researcher? e.g. personal  goals, reasons for doing the research |  |
|  |  |  | Participants were made aware of the purpose of the study. |
|  |  |  |  |
| Interviewer characteristics | 8 | What characteristics were reported about the inter viewer/facilitator?  e.g. Bias, assumptions, reasons and interests in the research topic |  |
|  |  |  | The interviewer was characterized as a chiropractor in pursuit of her PhD and was performing the interviews as part of her role as the research assistant. |
|  |  |  |  |
| **Domain 2: Study design** | | | |
| *Theoretical framework* | | | |
| Methodological orientation and Theory | 9 | What methodological orientation was stated to underpin the study? e.g. grounded theory, discourse analysis, ethnography, phenomenology,  content analysis |  |
|  |  |  | We used a qualitative descriptive approach as our methodological orientation. Inductive content analysis identified patterns and generated themes. |
|  |  |  |  |
| *Participant selection* | | | |
| Sampling | 10 | How were participants selected? e.g. purposive, convenience,  consecutive, snowball |  |
|  |  |  | Purposeful sampling |
|  |  |  |  |
| Method of approach | 11 | How were participants approached? e.g. face-to-face, telephone, mail,  email |  |
|  |  |  | Social media flyers online. |
|  |  |  |  |
| Sample size | 12 | How many participants were in the study? | 20 participants |
| Non-participation | 13 | How many people refused to participate or dropped out? Reasons? | One participant provided all demographic details but could not be reached for an interview |
| *Setting* | | | |
| Setting of data collection | 14 | Where was the data collected? e.g. home, clinic, workplace | Data was collected via the Zoom online platform with the participants either at their home or clinics. |
| Presence of non-  participants | 15 | Was anyone else present besides the participants and researchers? |  |
|  |  |  | No others were present during interviews. |
|  |  |  |  |
| Description of sample | 16 | What are the important characteristics of the sample? e.g. demographic  data, date |  |
|  |  |  | Sex, years of clinical experience, practice location and proportion of physical activity promotion. |
|  |  |  |  |
| *Data collection* | | | |
| Interview guide | 17 | Were questions, prompts, guides provided by the authors? Was it pilot  tested? | The interviews were semi structured with the use of questions. Tehe questions were pilot tested. |
|  |  |  |  |
| Repeat interviews | 18 | Were repeat inter views carried out? If yes, how many? | No repeat interviews were performed. |
| Audio/visual recording | 19 | Did the research use audio or visual recording to collect the data? | Both audio and visual recording were used to collect data. |
| Field notes | 20 | Were ﬁeld notes made during and/or after the inter view or focus group? | Methodological notes after, related to interview flow and adjusting. |
| Duration | 21 | What was the duration of the inter views or focus group? | Interviews ranged from 15-25 minutes. |
| Data saturation | 22 | Was data saturation discussed? | Data saturation and data sufficiency were discussed. |
| Transcripts returned | 23 | Were transcripts returned to participants for comment and/or correction? | Transcripts were returned to the participants for comment and/or correction. |

| **Topic** | **Item No.** | **Guide Questions/Description** | **Reported on**  **Page No.** |
| --- | --- | --- | --- |
|  |  |  |  |
| **Domain 3: analysis and**  **ﬁndings** | | | |
| *Data analysis* | | | |
| Number of data coders | 24 | How many data coders coded the data? | Two researchers coded the data with a third auditing transcripts to ensure the themes accurately reflected the data. |
| Description of the coding  tree | 25 | Did authors provide a description of the coding tree? |  |
|  |  |  | No description of the coding tree was provided. |
|  |  |  |  |
| Derivation of themes | 26 | Were themes identiﬁed in advance or derived from the data? | Themes were generated from the data itself. |
| Software | 27 | What software, if applicable, was used to manage the data? | Excel |
| Participant checking | 28 | Did participants provide feedback on the ﬁndings? | Participants did provided feedback on the findings. |
| *Reporting* | | | |
| Quotations presented | 29 | Were participant quotations presented to illustrate the themes/ﬁndings?  Was each quotation identiﬁed? e.g. participant number |  |
|  |  |  | Quotations were presented to illustrate sub themes, and were identified using a participant label (chiro XX) |
|  |  |  |  |
| Data and ﬁndings consistent | 30 | Was there consistency between the data presented and the ﬁndings? | There was consistency between the data presented and the ﬁndings. |
| Clarity of major themes | 31 | Were major themes clearly presented in the ﬁndings? | Major themes were clearly presented. |
| Clarity of minor themes | 32 | Is there a description of diverse cases or discussion of minor themes? | No description of diverse cases or discussion of minor themes was presented. |

Developed from: Tong A, Sainsbury P, Craig J. Consolidated criteria for reporting qualitative research (COREQ): a 32-item checklist for interviews and focus groups. *International Journal for Quality in Health Care*. 2007. Volume 19, Number 6: pp. 349 – 357

**Once you have completed this checklist, please save a copy and upload it as part of your submission. DO NOT include this checklist as part of the main manuscript document. It must be uploaded as a separate file.**
